# New electrocardiographic insights into left bundle branch block and the heart’s electrical position: Case series

**DOI:** 10.1016/j.hrcr.2025.03.009

**Published:** 2025-03-17

**Authors:** Rogelio Robledo-Nolasco, Elias Noel Andrade-Cuellar, Juan Carlos Solis-Gómez

**Affiliations:** Cardiac Electrophysiology, National Medical Center “November 20th,” ISSSTE, Mexico City, Mexico

**Keywords:** Left bundle branch block, Electrical axis, Heart’s electrical position, TAVR, Positive concordance, Negative discordance

**Key Teaching Points**

• The baseline electrical position of the heart is a critical factor influencing electrocardiographic changes following the development of left bundle branch block (LBBB) after transcatheter aortic valve replacement, with distinct patterns in both the intermediate and horizontal positions.
• In the intermediate electrical position without LBBB, the QRS and T wave axes fall within normal ranges, with positive concordance in leads II/III and aVL/aVF. In contrast, the horizontal position shows QRS and T wave axes slightly shifted to the left, with positive discordance in leads II/III and aVL/aVF.
• After LBBB develops, the intermediate position shows a mild leftward shift of the QRS and T wave axes, with partial loss of concordance in leads II/III and aVL/aVF. In the horizontal position, the QRS axis deviates markedly to the left, with total loss of concordance in leads II/III and aVL/aVF.

## Introduction

Transcatheter aortic valve replacement (TAVR) has become an established therapeutic option for patients with severe aortic stenosis who are at high or intermediate surgical risk.^1^^,^^2^ However, left bundle branch block (LBBB) remains a frequent complication following the procedure, carrying clinical implications such as heart failure, arrhythmias, and increased mortality.^3^^,^^4^ Recent studies propose stricter criteria to define a “true” LBBB, including a QRS duration exceeding 130–140 ms based on sex and the presence of notched morphologies in left-sided leads.^5^^,^^6^ Nonetheless, these criteria may not fully account for anatomic variability and the heart’s electrical position.

The heart’s electrical position, categorized as horizontal, intermediate, or vertical, reflects the spatial orientation of the ventricular depolarization vector and modifies the electrocardiographic appearance.^7^^,^^8^ In the context of LBBB after TAVR, evaluating the electrical position may provide insight into why some patients exhibit q waves in lateral leads or deeper QRS axis deviations. This article describes a series of 10 cases exploring how the baseline electrical position influences the manifestation and pattern of LBBB, with potential implications for risk stratification and patient monitoring.

## Case report

This case series included 10 patients who developed LBBB after TAVR; of these, 5 patients had an intermediate position and 5 patients had a horizontal position before LBBB. Baseline characteristics included a mean age of 75% ± 6 years, 60% male, 70% with systemic arterial hypertension, 40% with diabetes mellitus, and 10% with COPD. The mean ejection fraction was 54% ± 3%. Four patients received balloon-expandable prostheses, and 6 patients received self-expanding devices. No marked differences were noted between the 2 subgroups. None of the patients showed baseline conduction abnormalities on electrocardiogram (ECG). Serial ECGs were obtained: before TAVR, immediately afterward, at 24 hours, between days 2 and 3, at hospital discharge (approximately 5 days), and at 30 days, documenting the onset of LBBB and the baseline electrical position (intermediate or horizontal). In addition, electrocardiographic variables (QRS axis, T wave axis, PR interval, QRS duration, concordance/discordance in II/III and aVL/aVF) and clinical outcomes (progression to advanced atrioventricular [AV] blocks or pacemaker implantation) were assessed (Table 1).

**Table 1:** Baseline and follow-up electrocardiographic variables after post-TAVR LBBB

Parameters	Intermediate (n = 5)		Horizontal (n = 5)	
	Baseline	With LBBB	Baseline	With LBBB
QRS axis (°)	50	–20	–10	–60
T wave axis (°)	45	–10	0	+40
Concordance (+) II/III	5 (100%)	0 (0%)	0 (0%)	0 (0%)
Concordance (+) aVL/aVF	5 (100%)	3 (60%)	0 (0%)	0 (0%)
Concordance (–) II/III	0 (0%)	1 (20%)	0 (0%)	5 (100%)
Discordance (+) II/III	0 (0%)	5 (100%)	5 (100%)	0 (0%)
Discordance (+) aVL/aVF	0 (0%)	2 (40%)	5 (100%)	5 (100%)
qR pattern in I/aVL	0 (0%)	1 (20%)	1 (20%)	2 (40%)
Absence of q/Q in I/aVL	5 (100%)	4 (80%)	4 (80%)	3 (60%)
qR pattern in V5/V6	0 (0%)	0 (0%)	0 (0%)	1 (20%)
Absence of q/Q in V5/V6	5 (100%)	5 (100%)	5 (100%)	4 (80%)
QS in V1/V2	0 (0%)	2 (40%)	0 (0%)	3 (60%)
rS in V1/V2	3 (60%)	4 (80%)	3 (60%)	3 (60%)
TIDI in V5/V6 >45 ms	0 (0%)	5 (100%)	1 (20%)	4 (80%)
Baseline PR (ms)	160 (±8)	—	165 (±5)	—
Final PR (ms)	—	160 (±0)	—	185 (±15)
Baseline QRS duration (ms)	90 (±4)	—	88 (±3)	—
Final QRS duration (ms)	—	148 (±4)	—	160 (±8)
Pacemaker requirement	0 (0%)	0 (0%)	0 (0%)	0 (0%)

LBBB = left bundle branch block; TAVR = transcatheter aortic valve replacement; TIDI = Terminal Intrinsic Deflection Index.

### Intermediate electrical position

**Figure 1:**
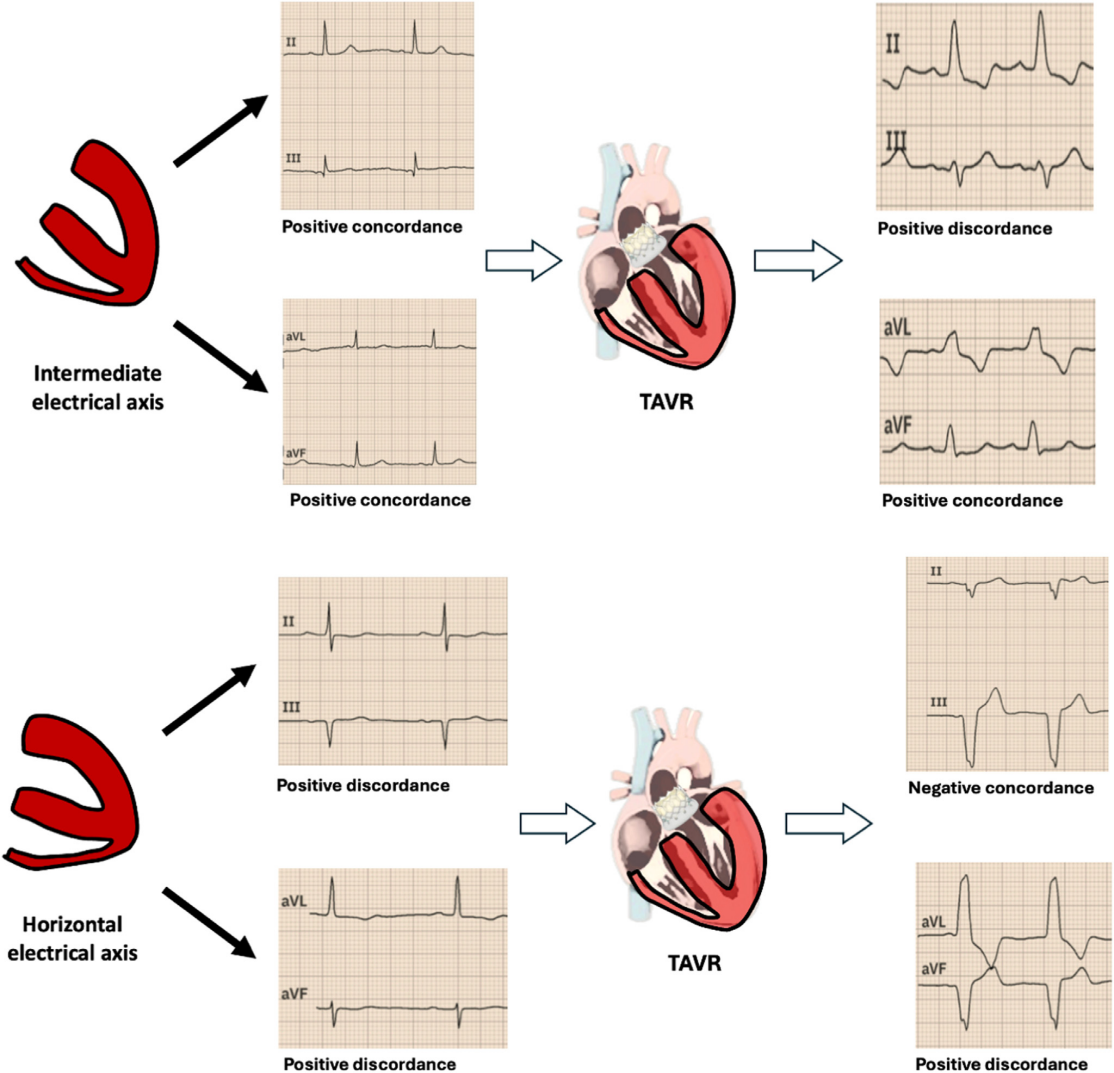
Novel electrocardiographic criteria for distinguishing intermediate and horizontal electrical positions in left bundle branch block.

In the intermediate electrical position (IEP) group (n = 5), the baseline QRS axis was +50°, with a T wave axis of +45°. There was no discordance in leads II/III or aVL/aVF, showing positive concordance in all of them. After LBBB developed, the QRS axis shifted to –20°, and the T wave axis shifted to –10°. Positive concordance in II/III was lost in most cases, with 1 patient (20%) displaying negative concordance in those leads. Sixty percent of patients maintained positive concordance in aVL/aVF. In 20% of patients, a qR pattern was observed in leads I/aVL and, likewise, in 20% in leads V5–V6. No patient required a pacemaker or progressed to advanced AV block (Table 1 and Figure 1).

### Horizontal electrical position

In the horizontal electrical position (H-EP) group (n = 5), the QRS and T wave axes were approximately –10° and 0°, respectively. All patients displayed positive discordance in leads II/III and aVL/aVF. Once LBBB occurred, the QRS axis deviated markedly to –60°, while the T wave axis shifted to +40°. None retained positive discordance in II/III (all 5 patients developed negative discordance in II/III), although 100% preserved discordance in aVL/aVF. A qR pattern was found in leads I/aVL in 40% of patients and in leads V5/V6 in 20% of patients. Two patients showed PR prolongation (20 and 40 ms), without progressing to advanced AV block (Table 1 and Figure 1).

## Discussion

In this case series, we investigated how the baseline electrical position of the heart influences the electrocardiographic changes that occur after LBBB in TAVR patients. Our findings indicate that intermediate and horizontal positions yield distinct ECG patterns. We focused on TAVR patients because LBBB is a frequent conduction abnormality in this setting, thus providing a suitable platform to examine the relationship between baseline electrical position and conduction modifications.^3^^,^^4^

In the group with an intermediate electrical position, the moderate shift of the QRS axis from +50° to –20°, along with the T wave axis from +45° to –10°, reflects altered ventricular activation affecting overall conduction.^1^^,^^4^ Partial preservation of positive concordance in aVL/aVF in 60% of these patients suggests that although left ventricular depolarization is abnormal (Figure 1), the activation pattern still retains some degree of organization.^5^^,^^6^

In contrast, the horizontal electrical position group exhibited more pronounced QRS axis shifts to –60° and T wave axis shifts to +40°. These changes were accompanied by complete loss of concordance in leads II/III (Figure 1) and a higher prevalence of qR patterns in leads I/aVL (40%) and V5/V6 (20%). Such findings suggest a more severe disruption of left ventricular conduction, likely associated with increased mechanical dyssynchrony. The reorientation of the depolarization vector toward a leftward and superior direction—typical of this group—may indicate a more significant delay in left ventricular activation.^6^^,^^8^

It is worth noting that classic LBBB ECG parameters, such as q waves in leads I and V5/V6 and a Terminal Intrinsic Deflection Index (TIDI) >45 ms, did not differ significantly between groups.^6^^,^^9^ This finding indicates that although LBBB induces substantial changes in electrical axis and concordance/discordance patterns, other ECG markers may be more influenced by anatomic or individual conduction characteristics than by the baseline electrical position.^7^^,^^9^^,^^10^

Our results suggest that the loss of concordance (especially in II/III) and extreme leftward QRS axis deviation might correlate with a significant delay in left ventricular activation. In keeping with previous reports, extreme left axis deviation has been linked to a reduced response to cardiac resynchronization therapy.^11^ Although we did not measure mechanical dyssynchrony directly in this study, the marked QRS axis shift in those with a horizontal position may indicate a greater conduction delay in that subgroup.

At 30-day follow-up, no patient progressed to complete AV block or required a pacemaker. However, 2 patients in the horizontal group experienced PR-interval prolongation, possibly suggesting increased conduction-system vulnerability in this subgroup. Larger cohorts with longer follow-up are warranted to better define the prognostic relevance of these findings.

Beyond QRS-duration or morphological criteria for diagnosing LBBB, evaluating the electrical position provides additional insights. Total loss of concordance in II/III and pronounced axis deviations may be linked to worse synchrony and, therefore, a higher risk of heart failure. Expanding the sample size in a prospective design could confirm these findings and establish stronger correlations with ventricular function, arrhythmic events, and the response to therapies such as resynchronization. This preliminary report serves as a starting point for further exploration of the interplay between electrical position and conduction abnormalities in TAVR patients.

## Conclusion

This case series demonstrates that the baseline electrical position of the heart significantly influences the electrocardiographic patterns arising after LBBB in TAVR patients. Those with an intermediate position showed less pronounced QRS and T-wave axis shifts, with partial preservation of concordance. In contrast, patients with a horizontal position exhibited extreme axis deviations, total loss of concordance, and higher rates of qR patterns. Recognizing these findings and integrating an analysis of the baseline electrical position could optimize risk stratification, guide monitoring, and potentially improve clinical management in this population.

## Disclosures

The authors have no conflicts to disclose.
